# No Improvement of Survival for Alveolar Rhabdomyosarcoma Patients After HLA-Matched Versus -Mismatched Allogeneic Hematopoietic Stem Cell Transplantation Compared to Standard-of-Care Therapy

**DOI:** 10.3389/fonc.2022.878367

**Published:** 2022-05-10

**Authors:** Sebastian Johannes Schober, Erika Hallmen, Florian Reßle, Hendrik Gassmann, Carolin Prexler, Angela Wawer, Irene von Luettichau, Ruth Ladenstein, Bernarda Kazanowska, Gustaf Ljungman, Felix Niggli, Olli Lohi, Julia Hauer, Bernd Gruhn, Thomas Klingebiel, Peter Bader, Stefan Burdach, Peter Lang, Monika Sparber-Sauer, Ewa Koscielniak, Uwe Thiel

**Affiliations:** ^1^ Department of Pediatrics, Children’s Cancer Research Center, Kinderklinik München Schwabing, School of Medicine, Technical University of Munich, Munich, Germany; ^2^ Klinikum der Landeshauptstadt Stuttgart gKAöR, Olgahospital, Stuttgart Cancer Center, Zentrum für Kinder-, Jugend- und Frauenmedizin, Pädiatrie 5 (Pädiatrische Onkologie, Hämatologie, Immunologie), Stuttgart, Germany; ^3^ Department of Pediatrics, Children’s Cancer Research Institute-S^2^IRP, St Anna Children’s Hospital, Medical University, Vienna, Austria; ^4^ Department of Pediatric Hematology/Oncology and BMT, Wroclaw Medical University, Wroclaw, Poland; ^5^ Department of Pediatric Hematology and Oncology, Department of Women’s and Children’s Health, Uppsala University, Uppsala, Sweden; ^6^ Pediatric Hematology and Oncology, University Hospital Zurich, Zurich, Switzerland; ^7^ Tampere Center for Child, Adolescent and Maternal Health Research, Faculty of Medicine and Health Technology, Tampere University and Tays Cancer Centre, Tampere University Hospital, Tampere, Finland; ^8^ Department of Pediatrics, Jena University Hospital, Jena, Germany; ^9^ Department of Pediatric Hematology and Oncology, Universitätsklinikum Frankfurt, Frankfurt, Germany; ^10^ Institute of Pathology, School of Medicine, Technical University of Munich, Munich, Germany; ^11^ Department of Pediatric Hematology and Oncology, Universitätsklinikum Tübingen, Tübingen, Germany; ^12^ Medizinische Fakultät der Universität Tübingen, Tübingen, Germany

**Keywords:** allogeneic stem cell transplantation, haploidentical, high-risk rhabdomyosarcoma, alveolar rhabdomyosarcoma (RMA) stage IV, matched-pair analysis, graft-versus-host disease, transplant-related mortality

## Abstract

**Background:**

Patients with stage IV alveolar rhabdomyosarcoma (RMA) have a 5-year-survival rate not exceeding 30%. Here, we assess the role of allogeneic hematopoietic stem cell transplantation (allo-HSCT) for these patients in comparison to standard-of-care regimens. We also compare the use of HLA-mismatched vs. HLA-matched grafts after reduced vs. myeloablative conditioning regimens, respectively.

**Patients and Methods:**

In this retrospective analysis, we compare event-free survival (EFS), overall survival (OS), and toxicity of HLA-mismatched vs. -matched transplanted patients in uni- and multivariate analyses (total: *n* = 50, HLA-matched: *n* = 15, HLA-mismatched: *n* = 35). Here, the factors age at diagnosis, age at allo-HSCT, sex, Oberlin score, disease status at allo-HSCT, and HLA graft type are assessed. For 29 primarily transplanted patients, three matched non-transplanted patients per one transplanted patient were identified from the CWS registry. Outcomes were respectively compared for OS and EFS. Matching criteria included sex, age at diagnosis, favorable/unfavorable primary tumor site, and metastatic sites.

**Results:**

Median EFS and OS did not differ significantly between HLA-mismatched and -matched patients. In the mismatched group, incidence of acute GvHD was 0.87 (grade III–IV: 0.14) vs. 0.80 in HLA-matched patients (grade III–IV: 0.20). Transplant-related mortality (TRM) of all patients was 0.20 and did not differ significantly between HLA-mismatched and -matched groups. A proportion of 0.58 relapsed or progressed and died of disease (HLA-mismatched: 0.66, HLA-matched: 0.53) whereas 0.18 were alive in complete remission (CR) at data collection. Multivariate and competing risk analyses confirmed CR and very good partial response (VGPR) status prior to allo-HSCT as the only decisive predictor for OS (*p* < 0.001). Matched-pair survival analyses of primarily transplanted patients vs. matched non-transplanted patients also identified disease status prior to allo-HSCT (CR, VGPR) as the only significant predictor for EFS. Here, OS was not affected, however.

**Conclusion:**

In this retrospective analysis, only a subgroup of patients with good response at allo-HSCT survived. There was no survival benefit of allo-transplanted patients compared to matched controls, suggesting the absence of a clinically relevant graft-versus-RMA effect in the current setting. The results of this analysis do not support further implementation of allo-HSCT in RMA stage IV patients.

## Introduction

Alveolar rhabdomyosarcoma (RMA) is a highly malignant pediatric soft tissue sarcoma entity, predominantly characterized by the oncogenic fusion gene forkhead box protein O1 (FOXO1) from chromosome 13 with either paired box protein 3 or 7 (PAX3, PAX7) from chromosome 2 or 1 [t(2;13)(q35;q14) or t(1;13)(p36;q14)] (1–3). Typical clinical manifestation of the primary disease includes extremities, the head and neck region, and other localizations (i.e., perineal and perianal) (4, 5). Unfavorable localization of the primary tumor, such as extremities, implicates a higher risk for relapse and poorer prognosis (6, 7). For non-metastatic RMA, estimated 5-year overall survival (OS) rate is 50%–65% (5, 8). With metastatic disease at diagnosis, 5-year-survival rates do not exceed 30% (6, 9). All rhabdomyosarcoma subtypes present with metastatic bone marrow (BM) involvement in approximately 6% of cases (10). In this disease constellation, 3-year event-free survival (EFS) is about 14% compared to 34% without BM disease (11, 12).

For metastasized disease and/or diagnosis at age > 10 years and/or bone/BM metastases, experimental therapeutic approaches currently under early clinical investigation can be considered CWS-guidance (version 1.6.1). Therefore, allogeneic hematopoietic stem cell transplantation (allo-HSCT) was implemented in some of these patients, hypothesizing the presence of a clinically relevant graft-versus-tumor effect (13, 14). It is unclear whether the use of allo-HSCT yields a survival benefit in the consolidation of prior complete response (CR) vs. non-remission at the time of transplantation as prospective studies are missing (15, 16).

Within the HD CWS-96 study, toxicity rates in patients treated with high-dose (HD) chemotherapy and autologous rescue vs. oral chemotherapy maintenance (OMT) were higher with significantly worse OS outcomes (17). The use of reduced-intensity conditioning regimens (RIC) prior to haploidentical HSCT was growingly implemented to enable a hypothesized graft-versus-RMA effect based on HLA disparity in the last decade.

In this retrospective analysis, we hypothesized that HLA-mismatched compared to HLA-matched allo-HSCT would increase survival rates in RMA patients, indicative of a graft-versus-RMA effect associated with HLA disparity. Furthermore, we hypothesized that a graft-versus-RMA effect would increase EFS and OS of transplanted- vs. matched non-transplanted RMA patients. Primary study objectives in both cases were EFS and OS.

## Patients and Methods

### Study Design and Data Acquisition

Data were retracted from the Cooperative Weichteilsarkom Studiengruppe (CWS) European soft tissue sarcoma registry (SoTiSaR) after approval by the principal investigators’ institutional ethical commission. For 50 RMA patients (stage III–IV), information concerning patient history and therapeutic interventions were used for further evaluation. All patients were diagnosed with RMA after the year 2000. Database update and last data collection was June 2020. RMA was confirmed by a local and expert pathologist, with genetic confirmation of PAX3/PAX7-FOXO1 fusions in 35 of 38 cases; 3 cases were fusion-negative. Molecular diagnosis was not done in 12 cases.

### Definitions

Engraftment was defined as absolute neutrophil count (ANC) ≥0.5 × 10^9^/L. Engraftment failure: ANC <0.5 × 10^9^/L by day +28 after allo-HSCT. When patients succumbed within ≤100 days after allo-HSCT, status of chronic GvHD could not be assessed. Further information on chronic GvHD was not available, due to limited recording in the past. Transplant-related mortality (TRM) was defined as any transplant-related death occurring after allo-HSCT without evidence of underlying disease or disease progression. Death of disease (DOD) was defined as any death related to underlying disease progression or relapse. Response was evaluated as follows: progressive disease (PD) was defined as tumor volume progression ≥50%, partial remission (PR) was defined as tumor volume reduction ≥50%, very good partial response (VGPR) was defined as tumor volume reduction ≥90% or persistence of unclear residuals upon imaging, and complete response (CR) was defined as complete disappearance of all visible disease. Measurement of tumor volume was done according to CWS guidance (1.6.1) using the formula V = π/6 × length × width × thickness. HLA-mismatched was defined as ≤9/10 differing HLA-I and -II alleles. In case of the comparison within HLA-mismatched versus HLA-matched setting, survival data were calculated from the time of 1st allo-HSCT until an event (i.e., relapse—EFS or TRM) or DOD for OS. For matched-pair analysis, survival was calculated from first therapeutic intervention until an event occurred (relapse, TRM, or DOD). Glucksberg criteria were used to grade GvHD.

### Patients

The study group consisted of a total of 50 transplanted patients, with 21 female patients (0.42) and 29 male patients (0.58). Median age at diagnosis was 14 years (range 0–24 years) with a median age at allo-HSCT of 14.0 years (range 0–24 years). Comparable to previously published analyses (18), patients were divided into two groups: HLA-mismatched (*n* = 35) and HLA-matched (*n* = 15). In the HLA-mismatched group, graft source was sibling in 2 patients, maternal in 17 patients, paternal in 15 patients, and both maternal and paternal in one patient. For HLA-matched patients, graft source was sibling in 14 (sister in 10/14 patients and brother in 4/14 patients). Patients qualified for allo-HSCT due to stage IV metastatic disease and/or age > 10 years and/or bone/bone marrow metastases at diagnosis or relapsed disease. Oberlin scores were used to assess further risk stratification (6). After conditioning therapy, 27/50 (0.54) patients were transplanted in CR or VGPR and 23/50 (0.46) patients were transplanted in PR or PD. Forty out of 50 (0.80) patients received primary allo-HSCT due to their very high-risk disease and/or lack of response to induction therapy. Allo-HSCT as relapse therapy was implemented in 10/50 patients (0.20). As a planned high-dose induction, 5/50 (0.10) received myeloablative chemotherapy and autologous HSCT (HLA-mismatched group = 4, HLA-matched group = 1) prior to allo-HSCT. Sex, age at diagnosis, age at allo-HSCT, primary site (favorable/unfavorable), bone or bone marrow involvement, stage, Oberlin score, eligibility for allo-HSCT, and disease status at allo-HSCT did not differ significantly in between groups A and B (**Table 1**). Four patients underwent multiple allo-HSCT, either due to relapse after 1st allo-HSCT (*n* = 2) or engraftment failure of the 1st allograft (*n* = 2). Mainly, transplants consisted of peripheral blood-derived stem cells (49 of 55 applied allografts, fraction 0.89), and 5/55 allografts (0.09, 1/55 source unknown) were derived from bone marrow. Treatment decisions were in concordance with approvals of institutional review boards and in accordance with the Helsinki Conference Declaration. Informed consent was signed by all patients or their legal guardians before initiation of therapy.

**Table 1:** Patient characteristics.

		HLA-mismatched	Fraction	HLA-matched	Fraction	*p*-value
**Total**	50	35		15		
**Sex**						
	F	12	0.34	9	0.60	0.13
	M	23	0.66	6	0.40	
**Age at diagnosis**						
	≤10	14	0.40	5	0.33	0.37
	>10 < 21	18	0.51	7	0.47	
	≥21	3	0.09	3	0.2	
**Age at allo-HSCT**						
	≤10	13	0.37	5	0.33	0.78
	>10 < 21	18	0.51	8	0.53	
	≥21	4	0.11	2	0.13	
**Primary site**						
	Favorable	8	0.23	6	0.40	0.48
	Unfavorable	27	0.77	8	0.53	
	Unknown	0	0.00	1	0.07	
**Bone or bone marrow involvement**						
	No	3	0.09	1	0.07	0.44
	Yes	30	0.86	12	0.80	
	Unknown	2	0.06	2	0.13	
**IRS group**						
	III	3	0.09	2	0.13	0.63
	IV	32	0.91	13	0.87	
**Oberlin score**						
	1	2	0.06	1	0.08	0.19
	2	5	0.16	6	0.46	
	3	13	0.41	2	0.15	
	4	12	0.38	4	0.31	
	n.a.	3		2		
**Eligibility for allo-HSCT**						
	Metastatic disease	29	0.83	11	0.73	0.46
	Relapse	6	0.17	4	0.27	
**Disease status at allo-HSCT**						
	CR/VGPR	19	0.54	8	0.53	0.69
	PR	13	0.37	7	0.47	
	PD	3	0.09	0	0.00	

F, female; M, male; CR, complete response; n.a., not applicable; VGPR, very good partial response; PR, partial response; PD, progressive disease; IRS, Intergroup Rhabdomyosarcoma Study; post-surgical group.

### Conditioning Regimens, Graft Manipulation, GvHD Prophylaxis, and Systemic Chemotherapies

Reduced-intensity conditioning (RIC) regimens (*n* = 37) were mostly based on fludarabine (*n* = 34) or clofarabine (*n* = 3), fludarabine monotherapy (*n* = 1), or in combination with either of the following: busulfan (BU, *n* = 3), cyclophosphamide (CYC, *n* = 7), etoposide (ETO, *n* = 3), melphalan (MEL, *n* = 31), thiotepa (TT, *n* = 34), or treosulfan (*n* = 1). Also, in most cases, either antithymocyte globuline (ATG, *n* = 11) or muromonab (OKT3, *n* = 18) was added. Total body irradiation (TBI) was performed in a minority of cases (*n* = 5). As for myeloablative condition regimens (MAC, *n* = 11), BU/CYC/ETO (*n* = 3), BU/TT/CYC (*n* = 1), BU/MEL/CYC (*n* = 1), BU/CYC/ATG (*n* = 1), CYC/TT/ATG (*n* = 1), CYC/treosulfan/ETO (*n* = 1), high-dose-ETO/ifosfamide (*n* = 1), and treosulfan/MEL (*n* = 1) were applied. TBI was given in 2 cases. Conditioning regimens were unknown in 2 cases.

Graft manipulation for HLA-mismatched grafts mainly consisted of CD3/CD19-depletion (*n* = 21, with TCRalpha/beta/CD19-depletion instead of CD3/CD19-depletion in 2 cases). CD3/CD19 depletions were combined with CD34 selection in 4 cases. CD34 selection was done alone in 3 cases, or in combination with T-cell depletion (*n* = 1). T-cell depletion alone was performed in 3 cases. Graft manipulation was unknown in 6 cases. Only one HLA-mismatched graft was not manipulated. HLA-matched grafts were not manipulated (*n* = 5), selected for CD34^+^ cells (*n* = 3), or CD3/CD19-depleted (*n* = 2). Status was unknown in 5 cases.

GvHD prophylaxis in the HLA-mismatched settings mainly contained mycophenolate mofetil (MMF, *n* = 27) alone or in combination with either corticosteroid, tacrolimus, post-transplant CYC, or ATG. Also, cyclosporine A (CSA) and methotrexate (MTX) were applied in/as combination. GvHD prophylaxis in the HLA-matched settings mainly composed of CSA (*n* = 11) alone or in combination with either MMF, tacrolimus, corticosteroid, MTX, sirolimus, or OKT3.

HLA-mismatched patients (*n* = 35) were treated according to protocols as follows: CWS96 (*n* = 2), CWS2000P (*n* = 9), CWS-IV-2002 (*n* = 6), SoTiSaR (*n* = 17), and one non-CWS regimen. HLA-matched patients (*n* = 15) were treated according to the following protocols: CWS96 (*n* = 6), CWS2000P (*n* = 5), SoTiSaR (*n* = 2), MMT-98 (*n* = 1), and one non-CWS regimen. Transplanted patients utilized for matched-pair analysis (*n* = 29) received systemic therapies according to CWS96 (*n* = 2), CWS2000P (*n* = 9), CWS-IV-2002 (*n* = 3), and SoTiSaR (*n* = 15). Non-transplanted patients (*n* = 87), who served as controls in the matched-pair analysis, were treated according to protocols as follows: CWS96 (*n* = 11), CWS2000P (*n* = 29), CWS-IV-2002 (*n* = 9), and SoTiSaR (*n* = 38).

### Matched-Pair Analysis

Within the CWS database, three matching patients per one transplanted patient were identified for the analysis. For 29 transplanted patients, who received primary allo-HSCT for their high-risk/progressive disease, matching non-transplanted pairs could be identified. Here, 22 transplanted patients received HLA-mismatched grafts and 7 patients received HLA-matched allografts. Matching criteria for further statistical analyses consisted of sex, age at diagnosis, favorable/unfavorable tumor site, and metastatic sites (locations). Primary systemic therapies for transplanted patients were either CEVAIE-based (*n* = 22) or VAIA-based (*n* = 7). CEVAIE is an intensive chemotherapy consisting of alternating courses of ifosfamide, vincristine, actinomycin-D (I^3^VA), carboplatin, epirubicin, vincristine (CVE) and ifosfamide, vincristine, and etoposide (I^3^VE, CWS guidance v1.6.1). VAIA is another chemotherapy regimen, currently used for the very high-risk group or metastatic stage in past protocols, implementing alternating cycles and combinations of vincristine, adriamycin, ifosfamide, and actinomycin-D. Primary systemic therapies for non-transplanted controls mainly included CEVAIE (*n* = 55) with O-TI/E (43/55) or were VAIA-based (*n* = 28) with O-TI/E (21/28), unknown in 4. The specific matching criteria and patients’ characteristics are summarized in **Table 4**.

### Statistical Analyses

End point values were recorded upon the last patient follow-up. Last database update was done in June 2020. Statistical analyses were performed using R studio 1.3.1093 (Public-benefit Corp), R for macOS X Cocoa GUI 4.1.0 (The R foundation for Statistical Computing) and Prism for macOS 9.1.2 (GraphPad Software). Fisher’s exact, Chi-square test, or Chi-square test for trend was applied to compare categorical variables in between study and control groups (for baseline characteristics). In multivariate analysis subdistribution hazard ratios (HR), standard errors, and confidence intervals (CI) are presented when appropriate. Survival probabilities were calculated using Kaplan–Meier curves and compared with a log-rank (Mantel-Cox) test. Cumulative incidence function and multivariate analyses were applied according to Scrucca et al. (19, 20). Statistical significance was attributed to *p*-values < 0.05.

## Results

### Engraftment and GvHD

Primary engraftment was stated in 45/50 (0.90) of cases; the rest experienced secondary engraftment. Median time to engraftment was 12 days (HLA-mismatched group, short mismatched: 13, HLA-matched group, short matched: 12). Incidence of acute GvHD in all analyzed patients was 0.85 (mismatched: 0.87 and matched: 0.80). No acute GvHD was reported in 15% of all cases (mismatched: 0.13 and matched: 0.20). Combined incidence with grade I–II acute GvHD was 0.66 (mismatched: 0.73 and matched: 0.50) indicating absent, or little to moderate toxicity in most of the cases. Incidence of extended chronic GvHD mounted up to 0.11 for all patients (mismatched: 0.09 and matched: 0.16). Status was unknown or not assessed in 10/50 cases for acute GvHD and 6/34 cases for chronic GvHD. Incidence of both acute and chronic GvHD did not differ significantly in between groups. More detailed information is provided in **Table 2**.

**Table 2:** Disease course after allo-HSCT.

		HLA-mismatched	Fraction	HLA-matched	Fraction	*p*-value
**Outcome**		35		15		
**Engraftment**						
	Primary	31	0.89	14	0.93	0.99
	Secondary	4	0.21	1	0.07	
**Acute GvHD**						
	Non	4	0.11	2	0.13	0.09
	Grade I–II	18	0.51	3	0.20	
	Grade II–III	3	0.10	2	0.13	
	Grade III–IV	5	0.14	3	0.20	
	Unknown	5	0.14	5	0.33	
**Chronic GvHD**						
	Non/limited	27	0.77	8	0.53	0.10
	Extended	3	0.09	2	0.13	
	Died before d+100	2	0.06	2	0.13	
	Unknown	3	0.09	3	0.20	
**Disease outcome: all (*n* = 50)**						
	Alive/other	4	0.11	5	0.33	0.10
	DOD	23	0.66	8	0.53	
	TRM	8	0.23	2	0.13	
**Disease outcome: stage IV (*n* = 45)**						
	Alive/other	3	0.09	3	0.23	0.23
	DOD	21	0.66	8	0.61	
	TRM	8	0.25	2	0.15	
**Disease outcome: primary allo-HSCT, stage IV (*n* = 40)**						
	Alive/other	3	0.10	3	0.27	0.29
	DOD	19	0.66	6	0.54	
	TRM	7	0.24	2	0.18	
**2-year EFS after allo-HSCT**			0.06		0.40	
(months)	median EFS	7		11		**0.001**
**2-year EFS after primary allo-HSCT**			0.07		0.27	
(months) median EFS		7		7		0.30
**OS after allo-HSCT (*n* = 50)**						
(months)	median OS	11		14		0.08
**OS after primary allo-HSCT, stage IV (*n* = 40)**						
(months)	median OS	15		12		0.60

Allo-HSCT, allogeneic hematopoietic stem cell transplantation; DOD, death of disease; EFS, event-free survival; GvHD, graft-versus-host disease; OS, overall survival; TRM, transplant-relatedmortality.

### Survival and Cause of Death—HLA-Mismatched Versus -Matched Grafts

**Figure 1:**
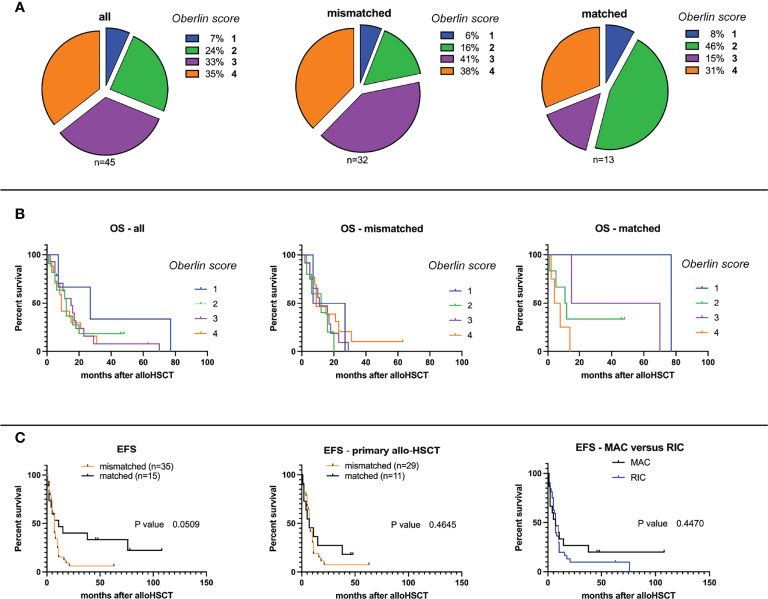
**(A)** Overall and event-free survival probabilities according to Oberlin score, HLA-mismatch/match, and conditioning regimen. Distribution of Oberlin risk factors (0–4) within study groups for HLA-mismatched and -matched analyses. **(B)** Overall survival (OS) probabilities after allogeneic hematopoietic stem cell transplantation (allo-HSCT) within study group population: all, HLA-mismatched, and HLA-matched (from left to right). **(C)** Event-free survival (EFS) probabilities after allo-HSCT for HLA-mismatched versus -matched grafts for all transplanted patients (left), primarily allo-transplanted patients (middle), and separated by myeloablative (MAC) or reduced-intensity conditioning (RIC, right).

Next, time intervals from the date of 1st allo-HSCT until occurrence of relapse, DOD, or TRM were compared. At the end of data collection, a total of 9/50 patients (0.18) were alive (or died of reasons unrelated to the primary disease or transplantation, labeled as “other”), 31 patients (0.61) died of primary disease (DOD), and for 10 patients, TRM (0.20) was reported, all without statistically significant differences in between groups. A more detailed overview of disease outcomes is given in **Table 2**. Mean follow-up time for patients grouped to alive/other was 45.8 months (mismatched: 24.5, matched: 62.8), 15.7 months for DOC (mismatched: 13.4, matched: 22.6), and 11.5 months for TRM (mismatched: 11.1, matched: 13.0), respectively. Although Oberlin risk factors were distributed without statistical significance in between HLA-mismatched and -matched patients, 0.79 of HLA-mismatched patients exhibited an Oberlin score ≥3, whereas only a fraction (0.46) in the HLA-matched group had a score ≥3 (**Table 1**, **Figure 1A**). Here, the 3 longest survivors (OS in months per patient: 130, 78, and 77) either had an Oberlin score of 1, or were not metastasized at diagnosis and all three received allo-HSCT as relapse therapy. Additionally, 2/3 had favorable primary tumor localization, 2/3 were younger than 10 years, and 2/3 had VGPR/CR at the time of allo-HSCT. As for the HLA-mismatched group, there was only one patient with a comparable distribution of risk factors and response to induction therapy, who survived for 21 months before DOD. As a consequence, 2-year EFS was significantly higher in the HLA-matched vs. -mismatched group (*p*-value = 0.001), with a median EFS of 7 months for mismatched and 11 months for matched (*p*-value = 0.051; median OS—mismatched: 11 months and matched: 14 months, *p*-value = 0.08) (**Table 2**). **Figure 1B** depicts survival curves according to Oberlin score. When analyzing the more homogeneous group of patients with primary allo-HSCT due to stage IV disease at diagnosis (*n* = 40), 2-year-EFS and median OS did not differ significantly (*p*-value = 0.46). Here, both groups showed a median EFS of 7 months and median OS for the mismatched group was 15 months versus 12 months for the matched group (*p*-value = 0.60). As most patients in the matched group received myeloablative conditioning (MAC, 0.86) and most HLA-mismatched cases received reduced-intensity conditioning (RIC, 0.91), we also precluded an effect due to different conditioning regimens (*p*-value = 0.45; **Figure 1C**) in a consecutive analysis.

**Figure 2:**
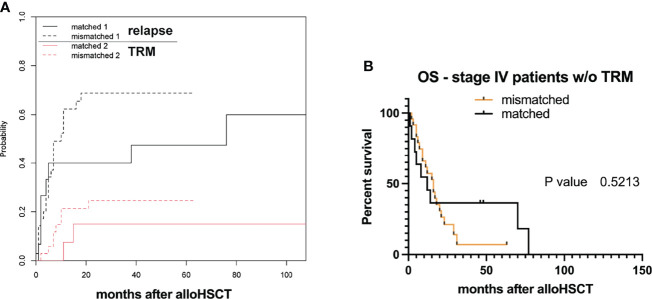
Transplant-related mortality (TRM) and survival. **(A)** Estimated cumulative incidence curves with relapse and transplant-related mortality (TRM) as competing events after allo-HSCT for HLA-mismatched versus -matched grafts. **(B)** Overall survival (OS) probabilities after allogeneic hematopoietic stem cell transplantation (allo-HSCT) within study group populations neglecting TRM (*w/o* without).

As TRM was relatively high, with a fraction of 0.20 in all analyzed cases, we furthermore performed a competing risk analysis by calculating the cumulative incidence function (CIF) for relapse, and separating it from TRM (as it will prevent emergence of later relapse) (19). Equality testing across groups revealed that there was no significant difference for relapse (*p*-value = 0.27) and TRM (*p*-value = 0.39). Differences in median OS were not observed even when excluding stage IV patients with TRM from the analysis (**Figures 2A, B**).

### Multivariate Regression Modeling

To confirm results of univariate analyses and to further identify additional factors decisive for survival, we performed multivariate regression modeling for competing risks data according to Scrucca et al. (20). Factors with possible influence on EFS and OS were analyzed, namely: age at diagnosis, age at allo-HSCT, Oberlin score (1–4, when applicable), disease status at allo-HSCT (VGPR/CR, PR, and PD), and graft type (HLA mismatch/match).

**Figure 3:**
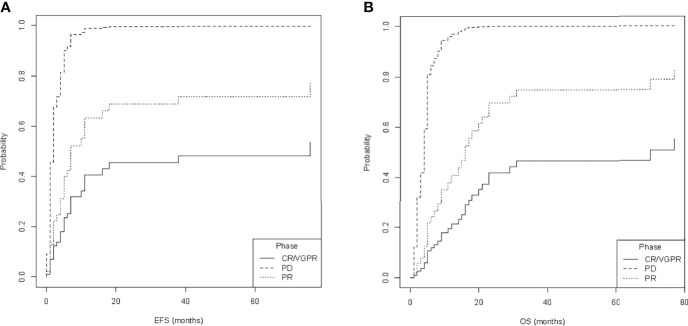
Estimated cumulative incidence of **(A)** relapse and **(B)** death, depending on the status at allogeneic hematopoietic stem cell transplantation (phase at allo-HSCT), namely, complete response or very good partial response (CR/VGPR), partial response (PR), and progressive disease (PD). Data of event-free survival (EFS) and overall survival (OS) were used for calculations.

Multiple models including different combinations of these covariates were fitted and compared (**Supplementary Table 1**). To select the best fit, the Bayesian information criteria (BIC) were applied. The ultimately selected model included disease status at allo-HSCT and Oberlin score. Only disease status correlated with outcome [*p*-values with respect to baseline (PD) for PR: *p*-value < 0.05 for EFS and OS; VGPR/CR: *p*-value < 0.001 for EFS and OS]. Therefore, CIF was calculated for relapse and death (DOD, TRM) depending on disease status at allo-HSCT (phase: VGPR/CR, PR, or PD, **Figure 3**). Here, disease status at allo-HSCT significantly correlated with outcomes. A summary of regression modeling for competing risks is provided in **Table 3**.

**Table 3:** Results of univariate and multivariate regression modeling for competing risks: model including all available covariates.

		HR	SE	95% CI		*p*-value
**EFS**						
Age at diagnosis		1.19	0.29	0.68	2.09	0.54
Age at allo-HSCT		0.76	0.29	0.43	1.34	0.35
Sex	M	ref				
	F	0.73	0.41	0.36	1.66	
Oberlin score		1.33	0.18	0.93	1.9	0.12
Status at allo-HSCT						
	PD	ref				
	PR	0.15	0.68	0.08	0.77	**0.016**
	VGPR/CR	0.06	0.57	0.03	0.39	**0.00058**
Graft type						
	HLA matched	ref				
	HLA mismatched	0.69	0.51	0.25	1.87	0.46
**OS**						
Age at diagnosis		1.35	0.30	0.75	2.40	0.32
Age at allo-HSCT		0.65	0.3	0.36	1.18	0.16
Sex	m	ref				
	f	0.57	0.45	0.23	1.37	0.21
Oberlin score		1.33	0.17	0.94	1.86	0.1
Status at allo-HSCT						
	PD	ref				
	PR	0.16	0.56	0.05	0.48	**0.001**
	VGPR/CR	0.07	0.63	0.02	0.23	**0.00002**
Graft type						
	HLA matched	ref				
	HLA mismatched	0.51	0.61	0.16	1.68	0.27

Allo-HSCT, allogeneic hematopoietic stem cell transplantation; CR, complete response; DOD, death of disease; EFS, event-free survival; F, female; M, male; OS, overall survival; PD, progressivedisease; PR, partial response; ref, reference; VGPR, very good partial response.

### Survival Analysis Compared to Matched Pairs

To address the question whether allo-HSCT provides survival benefits compared to standard-of-care therapy, we performed a matched-pair analysis. For 29 primary transplanted patients, matched non-transplanted pairs (3 non-transplanted per 1 transplanted patient) from CWS SoTiSaR were identified and compared for OS and EFS. Matching criteria included sex, age, favorable/unfavorable tumor site, and metastatic site. **Table 4** provides relevant patient characteristics according to matching criteria.

**Table 4:** Matched-pair analysis—patients summary.

	Allo-HSCT (*n* = 29)	Fraction	Controls (*n* = 87)	Fraction	*p*-value
**Sex**					>0.99
F	12	0.41	36	0.41	
M	17	0.59	51	0.59	
**Age at diagnosis**					0.28
≤10	*n* = 10		*n* = 30		
	Median 6.7		Median 6.0		
	Mean 6.8		Mean 6.1		
>10 <21	*n* = 15		*n* = 56		
	Median 15.6		Median 15.5		
	Mean 15.7		Mean 15.0		
≥21	*n* = 4		*n* = 1		
	Median 21.5		23		
	Mean 21.8				
**Primary tumor site**					0.15
Favorable	2		1		
Unfavorable	27		86		
**Metastatic site**					**0.04**
BM	5	0.17	6	0.07	
BM + other	5	0.17	5	0.06	
Bone	8	0.28	29	0.33	
Bone + other	6	0.21	20	0.23	
BM + bone	1	0.03	3	0.03	
BM + bone + other	4	0.14	24	0.28	
**2-year EFS**		0.24		0.15	
**Median EFS**	13		11		
**Median OS (months)**	22		18		0.67
**Deaths**	26/29	0.90	67/87	0.78	0.64

Allo-HSCT, allogeneic hematopoietic stem cell transplantation; BM, bone marrow; EFS, event-free survival; F, female; M, male; OS, overall survival.

For 2 primary transplanted patients, only 2 instead of 3 matched pairs were included for survival analysis. For the 4 transplanted patients aged ≥21 years, 11/12 matched patients were younger than 21 years. Of 2 transplanted patients presented with favorable tumor site at diagnosis, only one favorable matched control was identified.

As distribution in metastatic sites indicated a trend of unequal distribution in between study and control group (tested with Chi-square test for trend, with a tendency towards worse constellation of metastatic sites in the control group), which might influence survival analyses, regression modeling for competing risks was conducted accounting for metastatic site. No significant influence on survival was found (Wald test *p*-value = 0.08) (20).

**Figure 4:**
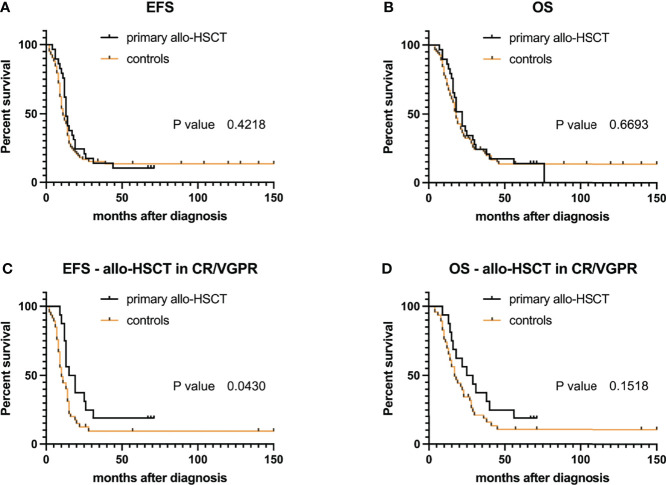
Event-free survival (EFS) and overall survival (OS) probabilities after allogeneic hematopoietic stem cell transplantation (allo-HSCT) compared to matched non-transplanted controls: for all 29 primary transplanted RMA patients **(A, B)**. **(C, D)** EFS and OS probabilities for patients with CR/VGPR at time of allo-HSCT (*n* = 16).

EFS probability for study and control groups did not differ significantly (*p*-value = 0.42) with a median EFS of 13 months for primary transplanted patients versus 11 months for controls (**Figure 4A**). Similar to EFS, probability to survive (i.e., OS) was not different (*p*-value = 0.67) with a median OS of 22 months for study group patients versus 18 months for controls (**Figure 4B**). As status at allo-HSCT was a condition for survival (CR or VGPR) in our previous analyses, we furthermore asked whether patients transplanted in CR/VGPR had a potential benefit from allo-HSCT compared to standard-of-care patients (controls). Indeed, respective patients show an increased survival probability for EFS (median 17 versus 10 months, *p*-value = 0.04). However, no differences were observed for OS (median 27 versus 17 months, *p*-value = 0.15, **Figures 4C, D**), even when neglecting cases of TRM (*p*-value = 0.12, graph not shown) or only focusing on those who received HLA-mismatched grafts (Kaplan–Meier curve comparison for EFS—*p*-value = 0.063 and for OS—*p*-value=0.09, graph not shown).

## Discussion

In this analysis, we hypothesized that (1) HLA-mismatched compared to HLA-matched allo-HSCT leads to survival rates in RMA patients, indicative of a graft-versus-RMA effect. Furthermore, we hypothesized that (2) a clinically relevant graft-versus-RMA effect improves survival in comparison to non-transplanted patients with similar risk factors. Data of patients diagnosed after 2000 were extracted from the CWS registry SoTiSaR. We sought to elaborate whether tumor control by allografts, and in this setting also HLA disparity (mismatched grafts), can be observed. As stage IV disease was the reason for allo-HSCT in most cases, its potential as part of the primary treatment should be evaluated.

In a previous retrospective analysis (15), evaluating the role of allo-HSCT for advanced rhabdomyosarcoma, we did not address the role of HLA-mismatched versus HLA-matched to induce a hypothesized graft-versus-tumor effect due to low frequency of haploidentical transplantations at that time. With better standardization and quality of *ex vivo* graft manipulation (e.g., CD3/CD19 depletion) (21), there has been a shift towards the use of haploidentical/mismatched grafts. In our analysis, we did not observe significant differences in between both study groups (mismatched vs. matched) in regard to EFS and OS. To minimize selection bias and competing risks, we performed multivariate analyses. Patient populations were small (mismatched *n* = 35, matched *n* = 15), imposing certain limitations in the statistical evaluation. Also, the mere fact that most mismatched grafts were T cell-depleted, whereas T-cell depletion was only done in 1/15 HLA-matched grafts, might have masked possible survival benefits in the HLA-mismatched group. An additional limitation constituted the heterogeneous treatment prior to transplantation. However, to our knowledge, this is the first systematic, albeit retrospective, evaluation of RMA patients comparing HLA-mismatched and -matched transplantation settings, as well as assessing a possible survival benefit compared to a non-transplanted control group.

In the present study, incidence of acute and chronic GvHD was comparable to other studies published for RMS (15, 22, 23) or EwS (18). Notably, TRM in our analysis (0.20) is higher than described for RMS by Merker et al. (22) (0.12; 17 of 25 studied patients with RMA) or Thiel et al. (15) (0.13; 23 of 30 studied patients with RMA) but did not differ significantly in between group A and B. The higher incidence of TRM, in comparison to earlier reports, might be due to a different and updated patient population, longer observation periods in our study, and differences among transplantation centers. Nonetheless, this retrospective analysis describes a relatively homogeneous population of 50 RMA patients. Considering TRM despite *ex vivo* graft manipulation is of utmost importance, although novel techniques for GvHD prophylaxis, such as post-transplant cyclophosphamide, were only implemented in one of the herein reported patients (21, 24). None of the patients with PD at allo-HSCT survived. This is also in line with published reports (15). In our analysis, response to induction therapy is the only reliable predictor for survival. Our data demonstrate that allo-HSCT in RMA patients is not associated with improved survival compared to matched controls (mainly treated with CEVAIE and OMT). Here, all transplanted survivors responded to induction therapy (6/9 patients had CR/VGPR). Additionally, 3/9 survivors were not metastasized at diagnosis, 3/9 had an Oberlin score of 2. Interestingly, one survivor with an Oberlin score of 4 and bone marrow disease was identified in the HLA-mismatched group. This constellation was recently described as fatal (12).

Although our matched-pair analysis revealed a higher probability of EFS for transplanted patients, it did not translate into increased OS. Hence, a clinically relevant graft-versus-RMA effect in our study population was not observed. Similar to high-dose chemotherapy followed by autologous rescue, patients treated with allo-HSCT might fare worse than OMT for IRS group/stage IV patients.

We previously described possible survival benefits from additional post-transplant immune-therapeutic strategies involving donor lymphocyte infusion (DLI) (25). In fact, one of the herein reported patients had BM disease at diagnosis with an Oberlin score of 4, responded very well to induction therapy (CR/VGPR), and survived without relapse at least 63 months after allo-HSCT. In this exceptional case, BM disease was controlled; hence, a graft-versus-tumor effect in this patient to control or maintain CR cannot be excluded.

Genetic engineering of T cells (e.g., with a chimeric antigen receptor, CAR) is a novel and promising approach, which may be implemented in patients with RMS in early clinical trials (NCT00902044). Indeed, in one case report utilizing a HER2 (human epidermal growth factor receptor 2)-specific CAR-T cell product, manufactured from autologous lymphocytes, repetitive application induced CR despite BM involvement. A second relapse after adoptive transfer was again treated with HER2-specific CAR-T cells combined with immune checkpoint blockade. This induced a third CR, which was ongoing for at least 20 months after cessation of T cells (26). Modified DLI with CAR-containing T cells might also be applied as an additive when considering allo-HSCT. This might be helpful to abrogate a T cell-hostile, immunosuppressive tumor microenvironment (27), due to additional cellular effectors in the graft [i.e., NK cells (28)], compared to CAR T-cell monotherapy.

In summary, in this retrospective study, only a subgroup of patients with good response at allo-HSCT survived. For stage IV patients, there was no survival benefit for either HLA-mismatched or HLA-matched transplant settings. Also, allo-transplanted patients with metastasis did not survive longer compared to matched controls, hinting at the absence of a clinically relevant graft-versus-RMA effect in our cohort. The results of this study do not support further implementation of allo-HSCT for stage IV RMA patients, especially with Oberlin scores ≥3, which is in accordance with many experts in the field (22, 29). We would like to emphasize though that this conclusion does not exclude the possible value of DLI in the treatment of RMA patients. The latter aspect may constitute a tool to initiate T-cell-mediated antitumor responses (25). This aspect, as well as the role of allo-HSCT, has not yet been elucidated sufficiently in prospective trials.

## Data Availability Statement

The data analyzed in this study is subject to the following licenses/restrictions: After ethical approval, relevant data were retracted from the Soft Tissue Sarcoma Registry (SoTiSaR) from the European Cooperative Weichteilsarkom Studiengruppe (CWS) containing sensitive information with potentially identifiable human data. Hence, relevant information supporting the conclusions of the manuscript are summarized in tables provided together with the manuscript. Requests to access these datasets should be directed to EH, erika.hallmen@olgahospital-stuttgart.de.

## Ethics Statement

The studies involving human participants were reviewed and approved by Ethikkommission der Technische Universität München. Written informed consent to participate in this study was provided by the participants’ legal guardian/next of kin.

## Author Contributions

Conception and design: SS and UT. Provision of study materials or patients: EH, AW, IL, RL, BK, GL, FN, OL, JH, BG, TK, PB, SB, PL, MS-S, and EK. Data analysis and interpretation: SS, EH, FR, HG, CP, EK, and UT. Manuscript writing: SS, HG, and UT. Final manuscript editing: SS, HG, CP, IL, TK, OL, MS-S, EK, and UT. All authors contributed to the article and approved the submitted version.

## Funding

This work was funded by a grant to SS and UT from the Wilhelm Sander-Stiftung (2021.007.1).

## Conflict of Interest

The authors declare that the research was conducted in the absence of any commercial or financial relationships that could be construed as a potential conflict of interest.

## Publisher’s Note

All claims expressed in this article are solely those of the authors and do not necessarily represent those of their affiliated organizations, or those of the publisher, the editors and the reviewers. Any product that may be evaluated in this article, or claim that may be made by its manufacturer, is not guaranteed or endorsed by the publisher.
