# Supplementary material for: No Improvement of Survival for Alveolar Rhabdomyosarcoma Patients After HLA-Matched Versus -Mismatched Allogeneic Hematopoietic Stem Cell Transplantation Compared to Standard-of-Care Therapy
Source: Front Oncol. 2022 May 10;12:878367. doi: 10.3389/fonc.2022.878367 (PMC9127413; doi:10.3389/fonc.2022.878367)
Supplement: Supplementary file 1 [file Table_1.docx]

Supplemental Table 1: Regression modelling of competing risks – model selection

| Tested models including the following covariates | | | | | | |  |  |
| --- | --- | --- | --- | --- | --- | --- | --- | --- |
|  |  |  |  |  |  |  |  |  |
|  | **AgeD** | **AgeHSCT** | **Oberlin** | **Sex** | **Phase** | **HLA_match** | **BIC diff** |  |
| mod0 |  |  |  |  |  |  | 3,87 |  |
| mod1 | x | x | x | x | x | x | 6,46 |  |
| mod2 |  |  |  | x | x | x | 7 |  |
| mod3 | x |  |  | x | x | x | 9,57 |  |
| mod4 |  |  | x | x | x | x | 8,85 |  |
| mod5 |  | x |  | x | x | x | 9,12 |  |
| mod6 |  |  |  |  | x |  | 0 |  |
| **mod7** |  |  | x |  | x |  | **1,21** |  |
|  |  |  |  |  |  |  |  |  |
|  | **Abbreviations and Explanations** | | | |  |  |  |  |
| mod | model |  |  |  |  |  |  |  |
| AgeD | age at diagnosis | |  |  |  |  |  |  |
| AgeHSCT | age at allo-HSCT | |  |  |  |  |  |  |
| Phase | disease status at allo-HSCT | | |  |  |  |  |  |
| HLA_match | HLA-mismatch or HLA match | | |  |  |  |  |  |
| BIC diff | >0<2 best model fit according to Scrucca et al.* | | | | | |  |  |
|  |  |  |  |  |  |  |  |  |
| *Scrucca L, Santucci A, Aversa F. Regression modeling of competing risk using R: an in depth guide for clinicians. Bone Marrow Transplant. 2010;45(9):1388-95. | | | | | | | | |
